# Correlates of loneliness in older adults in Shanghai, China: does age matter?

**DOI:** 10.1186/s12877-018-0994-x

**Published:** 2018-12-04

**Authors:** Fang Yang, Junan Zhang, Jianping Wang

**Affiliations:** Department of Social Work, School of Sociology and Political Science, Shanghai University, 99 Shangda Road, Baoshan District, Shanghai, 200444 China

**Keywords:** Loneliness, Young old, Old old, SUNS

## Abstract

**Background:**

Loneliness is a public health concern with serious health consequences in older adults. Despite a large body of research on the correlates of loneliness, little is known about the age group differences in the correlates. Given that the older adult population is heterogeneous, this study aims to examine the correlates of loneliness in older adults in Shanghai, and to explore how the correlates vary across different age groups.

**Methods:**

We used the Shanghai Urban Neighborhood Survey (SUNS) which was conducted in 2016 and 2017. The total sample size of older adults included in the analysis was 2770. Loneliness was measured using the sum of the 6 items derived from the De Jong Gierveld Loneliness Scale. Correlates include demographic variables, health conditions, social factors, and new media use. Regression analysis was used to examine the correlates of loneliness first in the whole sample, and then in the young old (60–79 years old) and the old old (80+ years old) separately.

**Results:**

The mean of loneliness score was 18.48 (SD = 5.77). The old old reported a higher level of loneliness than the young old. Variables, including age, living arrangement, marital status, education, health, family functioning, volunteering, square dancing, and new media use were found to be significant in the whole sample. Most of the significant correlates observed in the young old were identical to the findings reported for the total sample, with an exception for living arrangement. Self-rated health (SRH) and family functioning were two important correlates for the old old.

**Conclusions:**

Correlates of loneliness vary for the young old and the old old. The older adults at higher risk of loneliness deserve more attention and concern. Future interventions should be tailored to the young old and the old old to better help older adults alleviate loneliness and enhance their well-being.

## Background

Loneliness in older adults is a public health issue nowadays and deserves an increasing concern due to its negative effects in older adult population. Loneliness imposes a variety of health risks to older adults, such as cardiovascular health risk, declined cognitive function [1], increased morbidity and mortality [2–4] and poor mental health, including depression, anxiety, and sleeping disorder [5, 6], and health care utilization [7]. Loneliness is a unique clinical entity that deserves special and separate attention compared to other mental health issues, such as depression and anxiety [8]. The current study aims to examine the correlates of loneliness in the context of China, with an ultimate goal of improving the design and the intervention of alleviating loneliness among older adults.

Despite a sizeable literature on loneliness in Western societies, much less is known about loneliness in older adults in China, a country where family system and collectivism are greatly emphasized and one where dramatic socio- and demographic- changes have taken place in the past 40 years [9]. The culture and the family in which an individual develops is an important factor that affects their experience of loneliness [10, 11]. For instance, Dykstra found that older adults in more familialistic countries tend to be more lonely than those in individualistic societies [12]. Yum argued that loneliness is more about personal romantic expectations in some individualistic cultures, but is more related to social approval and social network in collective cultures [13]. Embedded in a collective culture, older adults may experience a higher risk of loneliness due to great changes, including increasing migration, smaller households, the weakening of traditional filial piety, and a rising number of empty-nest older adults. Moreover, people may be reluctant to acknowledge loneliness due to the Chinese cultural influence (e.g., face culture). The worse scenario is the unidentified loneliness, and the invisible loneliness without timely interventions leads to even undesirable consequences, as seeking help for emotional problems is less socially acceptable than for physical problems [14, 15]. The cultural difference and the characteristics of the aging population in China deserve special attention and research to provide a better understanding of the phenomenon of loneliness.

Although the definitions of loneliness are inconsistent in the literature, loneliness is usually defined as a subjective, negative, and distressing feeling due to the deficits in social contacts [16–20]. Due to the inconclusive definitions of loneliness, different measures have been used to assess loneliness, mainly including UCLA loneliness scale [19], De Jong Gierveld Loneliness scale [16, 21, 22], and a single item measure [9, 23]. The different measures make it difficult to compare the prevalence of loneliness of older adults in different countries or regions. Despite this, research suggests that there is an increase in the level of loneliness in older adults in China. For instance, Yang and Victor found that the prevalence of loneliness of older adults increased from 15.6% in 1992 to 29.6% in 2002 [9]. Similarly, Luo et al. found that there was a significant increase in the mean level of loneliness from 2002 to 2008 using a nation-wide survey [23]. Given the consequence of loneliness and the increase in the prevalence of loneliness in older adults in China, it is imperative to examine the loneliness level in older adults and the factors that are important to loneliness.

An overview of the current literature reveals that the correlates of loneliness in older adults include socio-demographics, health conditions, and psychosocial factors. The socio-demographic features associated with loneliness include being very old [18], gender [18], living arrangement [9, 24], and marital status [24, 25]. Regarding health factors, results imply that older adults with poor health in general tend to experience a higher level of loneliness, as poor health may hinder the maintenance of social contacts, and limit their daily activities that could be used to cope with loneliness [26]. Measures that have been used included self-rated health(SRH) [25, 27], chronic diseases, and ADL disabilities [27–29]. In addition, social factors were also found to be associated with loneliness, including social contacts [30, 31], social relationship satisfaction and social participation [9, 32].

In addition to these well-studied variables in the literature, new media use in older adults has been a hot topic in the communication research. Most studies have examined the characteristics of older adults who use new media or not, and the reasons why older adults do not use new media [33, 34]. Another line of research is about the impact of new media use on older adults’ well-being [35, 36],andquality of life [37]. Previous research suggests that the use of new media could be used to reduce loneliness in the older adults [38]. For instance, social media is a perfect platform for older adults and their family to communicate, especially when older adults usually do not share the same household with their adult children nowadays. Nevertheless, there is also research suggesting that the Internet use doesn’t necessarily benefit older adults in terms of loneliness [39]. In China, the ownership rate of smart-phones in middle-aged and older adults reached 95.6% [40], and more specifically the Internet penetration rate in Shanghai reached 74.1% [41]. New media has greatly impacted older adults’ way of living, such as reading news, entertainment, and social contact. Given the prevalence of new media in older adults, how it will impact older adults’ loneliness needs more research.

Despite a large number of literature on the correlates of loneliness, to the best of our knowledge, little is known about the age-group differences in the correlates of loneliness of older adults. Gerontological research suggests that the older adult population is not homogeneous, and there are two phases in late adulthood that are characterized by various qualities--namely the young old and the old old [42–44]. The young old (60–79), who are in their post-employment stage, are generally well functioning both physically and cognitively, and are actively socially engaged [42–44]. However, the old old (80+) often face increasing limitations to their independence due to more chronic conditions and health restrictions, and have a less-desirable psychological profile, and more death-related [42–44]. The different internal and external conditions associated with the two age groups may play a significant and differential role when older adults adapt to the age-related changes, such as social losses, or loneliness. Previous research on correlates of well-being and life attitudes actually indicates an age-differential pattern. For instance, Isaacowitz and Smith found that cognition and social relationships were significant predictors of positive affect only for the young old, but not for the old old [45]. Similarly, Jopp et al. supported age-differential correlates of valuation of life, such that health factors was especially stronger for the young old than for the old old [46]. These findings demonstrated that the correlates of loneliness might operate differently for the young old and the old old. For instance, the young old, who are relatively healthier, may compensate for being lonely by socially engaging in different activities, such as volunteering or square dancing. However, this might not be the case for the less healthier old old. Further empirical research is necessary to provide a more comprehensive picture of the extent to which the importance of the correlates of loneliness would vary in the two stages of later life.

We focused on older adults in Shanghai in the current study. China represents the largest aging population around the world. The population aged 60 and above accounts for 16.7% of the total population by 2016 [47]. Among the cities, Shanghai is the first aging city of China and has been an aging city since 1979, and the percentage of its aging population (60+) has reached 33.2% by the end of 2017 [48], which is almost twice higher than that of the whole nation. In addition to the high percentage of older adults, the aging population in Shanghai is also characterized by a high percentage of the oldest old and those living in “empty nest” households. The percentage of the oldest old (80+) has reached 16.4% among the older adults. The number of older adults living in “empty nest” households is more than 1 million, among of whom, 0.3 million are 80 years old and above. In this sense, Shanghai becomes an ideal place to examine older adults’ loneliness and its correlates. We believe such a study in Shanghai would shed light on the loneliness in other cities in China and would provide statistics to compare with other countries.

### The present study

This study aims to examine the correlates of loneliness in older adults in Shanghai, China and to explore whether there are age group differences in the correlates of loneliness. This study intends to address two gaps in the literature. First, the loneliness of older adults in China is relatively neglected. Research on this population’s loneliness would enrich our understanding of such a phenomenon. Second, the older adult population varies in terms of health and social characteristics, thus it is meaningful to identify the important correlates for the young old and the old old, and this would provide evidence on tailoring age-specific prevention and intervention programs.

## Method

### Data

We used the Shanghai Urban Neighborhood Survey (SUNS), which started in 2015 and was completed in 2017 by Center for Data and Urban Sciences (CENDUS). The data was sponsored by III project of sociology, Shanghai “GaofengGaoyuan” project. The data used Probability-Proportion-to-Size Sampling method with implicit stratification. In particular, the survey was conducted through three stages: the Primary Sampling Unit (PSU) was administrative subdistricts/towns; the Second Sampling Unit (SSU) was administrative villages/neighborhood communities; and the Third Sampling Unit (TSU)was households. Community-level survey was conducted in a randomly selected 537 communities among 5732 in the whole city. Household survey was conducted in a representative 180 communities, and all the individuals in the household sampled responded to the survey. SUNS is the largest city-wide survey that covers a variety of multi-level information (community, family, and individual-level), including sociodemographics, health-related information, attitudes towards family and community, social governance, and community information [41]. Inclusion criteria included: (1) participants were willing to respond the survey; (2) there were no language barriers; (3) participants have sufficient cognitive capacity to communicate. A sample of *N* = 8631 adults was interviewed. As our study focused on loneliness in older adults, only older adults aged 60 or above (*N* = 2770) were included in the current analysis. Please see research [49–51] for the details of the methodology.

### Measures

#### Loneliness

We used the De Jong Gierveld six-item scale for loneliness [16] to measure the overall loneliness. The scale has been translated into Chinese and proved to be valid and reliable measure of loneliness in Chinese older adults [21, 22]. Example item included “I experience a general sense of emptiness” and “I miss having people around”. Each item is measured on a 7-point Likert scale ranging from 1 = completely disagree to 7 = completely agree. Three items were firstly reversely coded, and then the sum score of the 6 items was obtained ranging from 6 to 42, with higher scores denoting higher level of loneliness. The reliability of the scale in this study was Cronbach’s α = 0.60.

#### Sociodemographic variables

The sociodemographic variables included age, gender(male vs. female), marital status (single, divorced, widowed vs. married), education (junior high, high, bachelor vs. primary school), living arrangement (living alone vs. living with other people), and these variables has been found to be related to loneliness in previous research [52].

#### Health factors

Health variables included: self-rated health (SRH), the number of chronic illnesses, and instrumental activities of daily living (IADL). SRH was measured by “in general, how would you rate your health?”, and it was measured on 5-point Likert scale, ranging from 1 = poor to 5 = very good. We used the number of the chronic illnesses to indicate their objective health status, with higher scores denoting more chronic illnesses. In addition, we used the IADL [53] to indicate older adults’ physical functioning, and the scale consisted of 7 items, e.g., food preparation, shopping, and transportation. The mean score of these items were used to indicate the level of disability, with higher scores denoting higher level of disability. The reliability of the scale in this study was Cronbach’s α = 0.90.

#### Social factors

We included family functioning, contact with neighbors, volunteering, playing mahjong, and square dancing. We used the 12-item General Functioning scale of the McMaster Family Assessment Device [54] to measure the overall emotional health and functioning of the family. Example items included “We confide in each other” and “There are lots of bad feelings in our family” [54]. Each item was measured on a 4-point Likert scale ranging from 1 = Strongly Agree to 4 = Strongly Disagree. Epstein et al. reported that the FAD general functioning scale had high reliability as well as good concurrent and predictive validity [54]. Six items were firstly reversely coded, and then the mean score of the 12 items was obtained, with higher score denoting better family functioning. The reliability of the scale in this study was Cronbach’s α = 0.74.

We asked participants to report the frequency of visiting or chatting with neighbors in the same community based on an option ranging from 1 = almost every day to 9 = almost never, and this item was reversely coded with higher scores denoting higher frequency. Participants also reported whether they volunteered in the previous year with an option of yes and no. In addition, participants reported the frequency of playing mahjong and square dancing based on a 9-point Likert scale ranging from 1 = almost every day to 9 = never. We reversely coded these two questions, so that higher score denoted higher frequency of playing mahjong and square dancing.

#### New media use

We asked participants to answer four new media use questions. We asked them “how often do you … online (using phone, the internet, or pad), including: read news, search health-related information, use QQ, WeChat, or other social media website, and entertain like playing games, watching videos, and listening to music”. The questions were measured on a 5-point Likert scale, ranging from 1 = never to 5 = almost every day. The average of the questions was used to indicate the frequency of new media use, with higher scores denoting more frequent new media use. The reliability of the scale in this study was Cronbach’s α = 0.90.

### Method of analysis

For variables with less than 2% missing values, we replaced missing values with the mode of those variables. For variables with more than 2% missing values, we used the multiple imputation to replace the missing values. We presented the descriptive statistics, and compared the differences of the variables in the young old and in the old old using t-tests and chi-square tests. Regression was then used to examine the correlates of loneliness in the whole sample first. Four models were designed. We included sociodemographic variables in Model 1, Model 2 added health-related variables, Model 3 added social factors, and Model 4 added new media use. In addition, we also examined how the correlates would vary across in the young old(60–79 years old; *N* = 2387) and in the old old (80+ years old; *N* = 383). The classifications of the two age groups vary in the literature, and we divide the aging population into two groups of 60–79 and 80+ based on the approach that has been widely used in previous studies in China [55, 56]. We tested the collinearity issues, and VIF showed that there was no such issue [57].

## Results

The mean age of the participants in this study was 69.72 years old (SD = 8.07). The majority of participants were current married (81.0%), with a highest education of junior high or lower (65%), and living with others (87.6%). In addition, 11.2% volunteered in the previous year (Table 1).

**Table 1:** Sample characteristics of the study

Variables	Total		Young old		Old old		Difference
	*N* = 2770		*N* = 2387		*N* = 383		
Sex							a
Male	48.80		49.4		45.7		
Female	51.20		50.6		54.3		
Hukou registration							
Urban	71.7		72.1		69.7		a
Rural	28.3		27.9		30.3		
Marital status							a^***^
Married	81.0		86.1		49.1		
Single	0.9		1.0		0.3		
Divorced	2.3		2.5		1.0		
Widowed	15.8		10.4		49.6		
Education							a^***^
Primary school or lower	34.9		30.2		64.5		
Junior high	29.2		32.3		10.2		
High	20.6		22		10.9		
Bachelor	15.4		15.5		14.4		
Living arrangement							a^***^
Living alone	12.4		10.6		24.0		
Living with others	87.6		89.4		76.0		
Volunteering	11.2		12.6		2.3		a^***^
	Mean	SD	Mean	SD	Mean	SD	
Age	69.72	8.07	67.23	5.24	85.22	4.57	b^***^
Self-rated health	2.16	0.86	2.17	0.87	2.08	0.81	b
IADL	1.27	0.62	1.17	0.46	1.90	1.01	b^***^
Chronic disease	2.09	1.79	1.99	1.74	2.68	2.00	b^***^
Family functioning	2.97	0.33	2.97	0.33	2.99	0.33	b
Chatting with neighbors	6.14	3.38	6.27	3.31	5.29	3.69	b^***^
Playing mahjong	1.96	2.34	1.98	2.36	1.79	2.23	b
Dancing	1.43	1.72	1.48	1.79	1.16	1.11	b^***^
New media use	1.87	1.27	1.98	1.32	1.17	0.61	b^***^
Loneliness	18.48	5.77	18.35	5.84	19.27	5.30	b ^**^

Note. **p < .01, ***p < .001. a. we used chi-square tests to compare the differences. b. we used t-tests to compare the differences. *IADL* instrumental activities of daily living

We also compared the differences of the key variables in the young old and in the old old (Table 1). Moreover, there were significant age-group differences with respect to marital status, education, living arrangement, volunteering experience, objective health indicators, contact with neighbors, playing mahjong, and new media use. Compared to the young old, the old old had a higher percentage of being widowed, were generally less educated, and were more likely to live with others. About 13% of the young old reported volunteering, but only 2.3% of the old old did. In addition, the old old reported more IADL and chronic diseases, and lower level of chatting with neighbors, square dancing, and new media use compared to the young old.

In terms of loneliness, the mean score was 18.48 (SD = 5.77). The loneliness level in the old old (M = 19.27, SD = 5.30) was significantly higher than that in the young old (M = 18.35, SD = 5.84).

### Regression results in the whole sample

As Table 2 shows, In Model 1, age, hukou, marital status, education, and living arrangement were significant. Compared to married older adults, those who were single and divorced experienced a higher level of loneliness (B = 2.71, *p* < .05; B = 3.37, *p* < .001, respectively), whereas the widowed older adults were not significantly different from their married counterparts. In terms of education, those with an education of high school and higher reported a lower level of loneliness compared to their less educated counterparts (high school: B = − 1.40, *p* < .001; bachelor: B = − 1.66, *p* < .001, respectively). Older adults living alone reported a higher level of loneliness than those living with others (B = 0.91, *p* < .05). In Model 2, SRH, IADL and chronic diseases (B = − 1.19, *p* < .001; B = 0.62, *p* < .01; B = 0.15, *p* < .05, respectively) were significantly correlated with loneliness, such that better health status was correlated with a lower level of loneliness. In Model 3, family functioning, chatting with neighbors, volunteering, and square dancing (B = − 4.31, *p* < .001; B = − 0.15, *p* < .001; B = − 0.88, *p* < .01; B = − 0.13, *p* < .05, separately) were negatively correlated with loneliness. Satisfactory family relationship, as well as higher frequency of contact with neighbors, volunteering, and dancing were correlated with a lower level of loneliness. In Model 4, more frequent new media use was related to a lower level of loneliness (B = − 0.59, *p* < .001).

**Table 2:** Correlates of loneliness in the older adults in Shanghai, total sample

	Model 1		Model 2		Model 3		Model 4	
	B	SE	B	SE	B	SE	B	SE
Age	0.05**	0.02	0.01	0.02	0.01	0.02	−0.01	0.02
Gender (female = 0)	0.26	0.22	0.45*	0.22	0.24	0.22	0.17	0.21
Urban Hukou (rural = 0)	−0.79**	0.28	− 0.65*	0.27	− 0.73**	0.26	− 0.52*	0.26
Marital status (married = 0)								
Single	2.71*	1.18	2.33*	1.15	2.39*	1.10	2.46*	1.10
Divorced	3.37***	0.75	3.10***	0.73	2.11**	0.70	2.27**	0.70
Widowed	0.25	0.38	0.21	0.37	0.29	0.36	0.34	0.35
Education (primary school or lower =0)								
Junior high	0.03	0.30	0.13	0.29	0.23	0.28	0.47	0.28
High	−1.40***	0.34	− 1.20***	0.34	−0.96**	0.32	−0.37	0.34
Bachelor	−1.66***	0.37	−1.36***	0.36	− 1.05**	0.35	−0.15	0.38
Living arrangement (living with others = 0)	0.91*	0.39	1.04**	0.39	0.92*	0.37	0.88*	0.37
Self-rated health			−1.19***	0.13	−0.93***	0.13	−0.91***	0.13
IADL			0.62**	0.19	0.33	0.19	0.28	0.19
Chronic disease			0.15*	0.06	0.17**	0.06	0.17**	0.06
Family functioning					−4.31***	0.31	− 4.16***	0.31
Chatting with neighbors					−0.15***	0.03	−0.15***	0.03
Volunteering (no = 0)					−0.88**	0.33	−0.86**	0.33
Playing mahjong					−0.07	0.04	−0.07	0.04
Dancing					−0.13*	0.06	−0.10	0.06
Media use							−0.59***	0.09
R^2^	4.5%		8.9%		16.7%		17.8%	

Note. *p < .05, **p < .01, ***p < .001. *IADL* instrumental activities of daily living

### Regression results by age groups

We further run the regression in the young old and in the old old. Results revealed that there were age-group differences in the correlates (Table 3). Most of the significant correlates observed in the young old were identical to the findings reported for the total sample, with an exception for living arrangement. Living arrangement was found to be significant only for the old old (B = 1.91, *p* < .01). In addition, variables that were significant for the young old lost their significant effect for the old old, including hukou, marital status, education, IADL, contact with neighbors, social participation, and new media use. However, SRH and family functioning remained significant for the old old.

**Table 3:** Correlates of loneliness in the older adults in Shanghai in the young old and in the old old

	Young old (60–79)				Old old (80+)			
	Model 1	Model 2	Model 3	Model 4	Model 1	Model 2	Model 3	Model 4
Age	0.06*	0.01	0.02	−0.01	0.12	0.17*	0.11	0.10
Gender (female = 0)	0.45	0.60*	0.34	0.28	−1.14	−0.86	−0.91	− 0.87
Urban hukou (rural = 0)	−0.68*	− 0.51	−0.64*	− 0.43	−0.88	− 0.86	−0.72	− 0.72
Marital status (married = 0)								
Single	2.78*	2.27	2.36*	2.42*	4.42	4.53	3.22	3.12
Divorce	3.99***	3.71***	2.68***	2.79***	−1.40	−1.04	−2.39	−2.48
Widow	0.90	0.92*	0.86*	0.88*	−.1.34*	−1.26	−0.76	−0.79
Education (primary school or lower =0)								
Junior high	−0.13	−0.01	0.16	0.44	−0.07	0.03	−0.06	−0.11
High	−1.74***	−1.51***	− 1.17**	−0.53	0.77	0.81	0.29	0.53
Bachelor	−1.95***	−1.61***	− 1.19**	−0.22	−0.83	− 0.53	−0.99	− 0.54
Living arrangement (living with others = 0)	0.40	0.59	0.58	0.57	1.91**	1.76*	1.45*	1.43*
Self-rated health		−1.15***	−0.90***	−0.88***		−1.45***	− 1.09**	− 1.06**
IADL		1.19***	0.85**	0.72**		−0.15	− 0.29	− 0.34
Chronic disease		0.12	0.14*	0.15*		0.18	0.18	0.19
Family functioning			−4.25***	−4.10***			−3.94***	−3.92***
Chatting with neighbors			−0.16***	−0.15***			−0.14	−.15*
Volunteering			−0.92**	−0.93**			2.23	2.48
Playing mahjong			−0.06	−0.07			−0.12	−0.13
Dancing			−0.14*	−0.11			0.13	0.10
Media use				−0.56***				−0.85
R^2^	4.9%	9.5%	17.1%	18.2%	5.1%	10.8%	18.6%	19.3%

Note. *p < .05, **p < .01, ***p < .001. *IADL* instrumental activities of daily living

## Discussion

The present study represents a first attempt to investigate loneliness in older adults of Shanghai, China, with a particular attention on the similarities and differences in the correlates between the young old and the old old in the backdrop of emerging socio-demographic features. We found that the old old reported a higher level of loneliness, and health status and social factors were important correlates of loneliness. In addition, analyses revealed an age-differential pattern, such that SRH and family functioning were important for both the young old and the old old, whereas marital status, education, social participation, and new media use were significant only for the young old.

We found that 4 to 39% of the participants agreed with at least one of the six items of the De Jong Gierveld loneliness scale, which suggests that the level of loneliness is not very high as expected, but still a small proportion of the sample acknowledge the feelings of loneliness. This finding challenges the often-held notion that older adults are lonely. There are several possible reasons. First, older adults may be reluctant to acknowledge the feeling of being lonely, as direct admission possibly indicates a lack of a satisfactory family relationship and in turn compromise their self-worth. Second, some older adults who live alone may have the necessary resources to choose such a living arrangement to pursue a sense of autonomy [58, 59]. Especially for the young old, living alone is not a significant risk factor of loneliness and this finding implied that living alone may not necessarily cause loneliness, instead it might be a personal choice out of autonomy and an indication of independence. More research is needed to explore the reasons behind such a phenomenon. More importantly, we noticed that those who were older, with rural hukou, single and widowed, less educated, living alone, in poorer health, with poorer family functioning, less socially engaged, and less frequent in using new media tend to experience a higher level of loneliness, and this suggests a “disadvantage accumulation” phenomenon. It is imperative to understand the meaning, reasons, and level of suffering implied on those feelings of loneliness, and to design multi-factorial interventions that target the deficits for older adults at high risk of loneliness.

Consistent with previous research, the mean level of loneliness was higher in the old old than in the young old [12, 18]. The old old experience more physical limitations and more social losses, and their expectations for social contacts may be difficult to fulfill, thus they experience higher loneliness than their young old counterparts. Regarding gender differences, we did not find significant results. This is in line with previous research implying that gender alone may not be directly related to loneliness, but rather interacts with other factors, such as marital status and living arrangement [18]. In addition, compared to married older adults, those who were single and divorced reported a significantly higher level of loneliness, whereas the widowed older adults were not significantly different from their married counterparts. Most of older adults’ support needs are satisfied by their spouses, being single or divorced implies an absence of such an intimate relationship, thus leading to more loneliness [60]. However, widowhood might be more of a normative state than being single or divorced for older adults [61], and this state imposes a less impact on loneliness, especially when they have a satisfactory family relationship. Moreover, more educated older adults tend to experience less loneliness, because they generally have better coping abilities and more resources to enrich their life and combat loneliness [62]**.** Compared to other living arrangements, those living alone reported a higher level of loneliness, and this is consistent with previous findings [63].

In terms of health factors, the three health indicators in the study were all significant, such that better health is associated with a lower level of loneliness. The loss of social contacts is part of the aging process, and poorer health further imposes obstacles for older adults to socialize, to effectively communicate with others, and to participate in social activities, all of which could be used to cope with loneliness [26, 64]. Less healthy older adults may also possess less resilience and lower self-efficacy to establish and maintain social contacts. Thus older adults in poorer health are at higher risk of loneliness.

In terms of social factors, humans are social creatures and a higher level of social integration and connections are protective factors of loneliness. Helping those in need and maintaining engagement with others is two important feature of volunteering, and it is a powerful antidote to loneliness because it gives people the opportunities to be needed [65]. In Shanghai, there are about 2.6 million registered volunteers, among whom 34% are over 60 years old by 2016 [66], and being a volunteer serves as a meaningful buffer for older adults’ loneliness. In addition, as there is an increase in the number of empty-nest older adults, neighbors are more easily available due to the proximity, and would be especially important for older adults’ daily contacts, thus associating with a lower level of loneliness [18].

An important question we examined in the current study is the age-group differences in the correlates of loneliness. Results revealed that SRH and family functioning are two very important correlates for both the young old and the old old. SRH is a global measure of health status [67], and has been found to predict a variety of outcomes, e.g., mental health, mortality [68], and loneliness [69]. Even though SRH is influenced by objective health, there is evidence suggesting that the correspondence between chronic diseases and SRH generally declines with age [70]. The old old could still have a positive SRH despite their poor health, and this opens up the opportunities for the intervention to improve older adults’ SRH [71]. In addition, family functioning is a very strong protector of loneliness. In the context of China where the culture greatly emphasized family system, individuals experience more intimate connections from family members, have more emotional communication, have higher mutual trust, and are better able to adapt to changes or hardships in a well-functioning family, all of which could minimize loneliness [3].

It is interesting that some of the significant factors for the young old lost their significance for the old old. Variables, including marital status, education, IADL, contact with neighbors, volunteering, and square dancing were no longer significant for the old old. These differences might be largely due to the differing qualities in these two age groups. The demographic variables are no longer significant for the old old, perhaps loneliness in the old old is more of a result of internal factors, such as how they view their health and family relationship [46]. The worse health status might be more of a normative phenomenon for the old old, whereas worse health status is an “off-time” event for the young old, thus leading to a significant effect of IADL and chronic illnesses only for the young old [72]. Healthier young old might benefit more from the social participation, whereas poorer health attenuates the beneficial effect of social engagement for the old old. It is also possible that the level of social participation in the old old does not reach the minimal level, beyond which social participation can be effective for alleviating loneliness. More importantly, new media use was associated with lower loneliness for the young old, but not for the old old. Perhaps the young old who are relatively healthier are more affected by the pervasive new media and are more capable of using the new media to communicate with others, to read what they are interested in, to entertain on the new media than the old old [33, 34].

This study has meaningful implications for interventions of alleviating loneliness. First, our study challenges the negative stereotype that older adults are lonely, actually the majority of the older adults reported being not lonely. Nevertheless, those who reported being lonely and those with higher risk of loneliness deserve more concern. Second, age-group differences were found for the correlates of loneliness, and age-tailored interventions are needed to better help older adults prevent or combat loneliness. For instance, interventions addressing SRH and family functioning are appreciated for both age groups, and those creating more opportunities for the young old to volunteer also could be helpful. Third, in an era of new technology, we could take advantage of new media to help them enrich their life and better communicate with their family or friends, such as teaching older adults how to use new media (e.g., Wechat) to chat with family or friends. Study suggested that contemporary information and communication technologies have the potential to prevent or reduce the social isolation of older adults [73]. Lastly, future interventions would be beneficial using a life-course perspective. Targeting risk factors at earlier stages of life could also prevent loneliness at later life stages.

There are several limitations that should be noted. First, the study is based on a cross-sectional dataset, thus the causal relationship among the variables cannot be established in the study. Future research is needed to examine what factors predict the onset and the alleviation of loneliness using longitudinal data, thus providing more robust evidence for interventions. Second, other factors that could possibly influence loneliness are not considered given the current information of the data, such as personality and community-level factors. It would benefit if future research could incorporate multi-level factors to provide an overarching picture of loneliness of older adults. Third, participants included in the study were only community-dwelling older adults. The results may not be generalized to those who live in the institutions.

## Conclusions

Our study examined correlates of loneliness, and as far as we know, and was the first attempt to explore age-group differences in loneliness of older adults in Shanghai, China, where population aging displays its unique features. Results revealed that loneliness in older adults does not prevail as the negative aging stereotype suggests. Older adults who are living alone, single and divorced, less educated, in poorer health, less socially engaged, less satisfied with family relationship, and less frequent in using new media are at a higher risk for loneliness. In addition, different important correlates of loneliness in the young old and in the old old are identified, such that some essential factors in the young old lost their significance in the old old. Future interventions should be tailored to the young old and the old old to better help older adults alleviate the loneliness and enhance their well-being.

## Abbreviations

IADL: Instrumental activities of daily living
SRH: Self-rated health
